# Gastrointestinal Bleeding, but Not Other Gastrointestinal Symptoms, Is Associated With Worse Outcomes in COVID-19 Patients

**DOI:** 10.3389/fmed.2021.759152

**Published:** 2021-10-13

**Authors:** Hongxin Chen, Zhenhua Tong, Zhuang Ma, Li Luo, Yufu Tang, Yue Teng, Hao Yu, Hao Meng, Chengfei Peng, Quanyu Zhang, Tianyi Zhu, Haitao Zhao, Guiyang Chu, Hongyu Li, Hui Lu, Xingshun Qi

**Affiliations:** ^1^COVID-19 Study Group, General Hospital of Northern Theater Command, Shenyang, China; ^2^Department of Gastroenterology, General Hospital of Northern Theater Command, Shenyang, China; ^3^Postgraduate College, Liaoning University of Traditional Chinese Medicine, Shenyang, China; ^4^Section of Medical Service, General Hospital of Northern Theater Command, Shenyang, China; ^5^Department of Respiratory Medicine, General Hospital of Northern Theater Command, Shenyang, China; ^6^Department of Infectious Diseases, Wuhan Huoshenshan Hospital, Wuhan, China; ^7^Information Section of Medical Security Center, General Hospital of Northern Theater Command, Shenyang, China

**Keywords:** coronavirus disease 2019, severe acute respiratory syndrome coronavirus 2, gastrointestinal symptoms, prevalence, outcomes

## Abstract

**Background:** Patients with coronavirus disease 2019 (COVID-19) can present with gastrointestinal (GI) symptoms. However, the prevalence of GI symptoms and their association with outcomes remain controversial in COVID-19 patients.

**Methods:** All COVID-19 patients consecutively admitted to the Wuhan Huoshenshan hospital from February 2020 to April 2020 were collected. Disease severity and outcomes were compared between COVID-19 patients with and without GI symptoms. Logistic regression analyses were performed to evaluate the association of GI symptoms with the composite endpoint and death in COVID-19 patients. A composite endpoint was defined as transfer to intensive care unit, requirement of mechanical ventilation, and death. Odds ratios (ORs) with 95% confidence intervals (CIs) were calculated.

**Results:** Overall, 2,552 COVID-19 patients were included. The prevalence of GI symptoms was 21.0% (537/2,552). Diarrhea (8.9%, 226/2,552) was the most common GI symptom. Patients with GI symptoms had significantly higher proportions of severe COVID-19 and worse outcomes than those without. Univariate logistic regression analyses demonstrated that GI symptoms were significantly associated with the composite endpoint (OR = 2.426, 95% CI = 1.608–3.661; *P* < 0.001) and death (OR = 2.137, 95% CI = 1.209–3.778; *P* = 0.009). After adjusting for age, sex, and severe/critical COVID-19, GI symptoms were still independently associated with the composite endpoint (OR = 2.029, 95% CI = 1.294–3.182; *P* = 0.002), but not death (OR = 1.726, 95% CI = 0.946–3.150; *P* = 0.075). According to the type of GI symptoms, GI bleeding was an independent predictor of the composite endpoint (OR = 8.416, 95% CI = 3.465–20.438, *P* < 0.001) and death (OR = 6.640, 95% CI = 2.567–17.179, *P* < 0.001), but not other GI symptoms (i.e., diarrhea, abdominal discomfort, nausea and/or vomiting, constipation, acid reflux and/or heartburn, or abdominal pain).

**Conclusion:** GI symptoms are common in COVID-19 patients and may be associated with their worse outcomes. Notably, such a negative impact of GI symptoms on the outcomes should be attributed to GI bleeding.

## Introduction

Severe acute respiratory syndrome coronavirus 2 (SARS-CoV-2) causes coronavirus disease 2019 (COVID-19) and has resulted in a global pandemic (1). Till August 12, 2021, there have been 200,644,849 confirmed cases of COVID-19 and 4,323,139 deaths.^1^ There is a wide clinical spectrum of COVID-19, ranging from asymptomatic infection, mild symptoms, to critical status. The most common clinical symptoms are fever, cough, and shortness of breath (2). COVID-19 patients can also present with gastrointestinal (GI) symptoms, such as diarrhea, abdominal pain, nausea, and vomiting (3). The prevalence of GI symptoms is heterogeneous among studies. A study conducted in China reported that the prevalence of GI symptoms was about 11% (4), while another study conducted in the United States reported that more than 61% of COVID-19 patients had GI symptoms (5). On the other hand, the influence of GI symptoms on the outcomes of COVID-19 patients remains controversial among studies. Some studies suggested that patients with GI symptoms had a higher risk of acute respiratory distress syndrome, mechanical ventilation, admission to intensive care, and death than those without (6, 7). By contrast, other studies found that patients with GI symptoms had a similar or even lower risk of mechanical ventilation and/or death than those without (8, 9). More notably, it remains unclear about which type of GI symptoms truly affects the outcomes of COVID-19 patients. Herein, we conducted a retrospective study to further explore the prevalence of GI symptoms in COVID-19 patients and analyze the association of GI symptoms with their outcomes with an emphasis on various types of GI symptoms.

## Materials and Methods

### Ethics

The study protocol was reviewed and approved by the Medical Ethical Committee of the General Hospital of Northern Theater Command with an approval number [Y (2021) 059] and performed according to the Declaration of Helsinki.

### Study Design

In this study, we retrospectively reviewed the medical records of 3,041 patients who were diagnosed as COVID-19 and consecutively admitted to the Wuhan Huoshenshan hospital from February 2020 to April 2020. Notably, this hospital was established by Chinese government to treat COVID-19 patients at the beginning of the SARS-CoV-2 outbreak in February 2020, and was closed after the epidemic was effectively controlled in April 2020. The exclusion criteria were as follows: (1) patients with hepatobiliary diseases, which mainly included hepatitis, liver cirrhosis, cholecystitis, and gallstones; (2) patients with a recent history of GI diseases or symptoms before admission, which mainly included esophagitis, gastritis, enteritis, peptic ulcer, chronic diarrhea, and constipation; (3) patients with a history of abdominal surgery, which mainly included cesarean section, hysterectomy, appendectomy, and cholecystectomy; (4) patients with nephrolithiasis; and (5) major clinical data were lacking.

### Data Collection

The following data was collected from electronic medical records: demographics (i.e., age and sex), COVID-19 symptoms (i.e., fever, cough, fatigue and/or myalgia, dyspnea, chest distress and/or shortness of breath, and expectoration), GI symptoms, comorbidities (i.e., diabetes, cardiovascular disease, cerebrovascular disease, chronic renal disease, chronic respiratory disease, and malignant tumor), laboratory tests at admission [i.e., hemoglobin (Hb), white blood cells (WBC), platelet count (PLT), total bilirubin (TBIL), aspartate aminotransferase (AST), alanine aminotransferase (ALT), alkaline phosphatase (AKP), gamma glutamyl transpeptidase (GGT), albumin, d-dimer, and prothrombin time (PT)], severity of COVID-19 at admission, major treatments for COVID-19 during hospitalization [i.e., antivirals, antibiotics, corticosteroids, traditional Chinese medicines, and extracorporeal membrane oxygenation/continuous renal replacement therapy (ECMO/CRRT)], and major in-hospital outcomes [i.e., requirement of mechanical ventilation, transfer to intensive care unit (ICU), and death].

### Definitions

GI symptoms were defined as the occurrence of at least one of the following GI symptoms during the course of COVID-19: diarrhea, abdominal discomfort, nausea and/or vomiting, constipation, acid reflux and/or heartburn, abdominal pain, and GI bleeding.

According to the New Coronavirus Pneumonia Prevention and Control Program published by the National Health Commission of China (Provisional, 7th Edition Revision), the severity of COVID-19 was classified as mild, moderate, severe, and critical type (10). Mild type was defined as mild clinical symptoms without any imaging evidence of pneumonia. Moderate type was defined as fever, respiratory symptoms, and imaging evidence of pneumonia. Severe type was defined as any one of the following criteria: (1) respiratory distress with respiratory rate>30 breaths per minute; (2) oxygen saturation (SpO_2_) < 93% in the resting state; (3) arterial partial pressure of oxygen (PaO_2_)/fraction of inspiration oxygen (FiO_2_) ≤ 3 00 mmHg. Critical type was defined as any one of the following criteria: (1) respiratory failure requiring mechanical ventilation; (2) shock; (3) other organ failures requiring ICU monitoring and treatment.

The composite endpoint was defined as transfer to ICU, requirement of mechanical ventilation, and death (11).

### Outcomes

Major outcomes included the prevalence of various GI symptoms in patients with COVID-19 and the association of GI symptoms with the composite endpoint and death.

### Statistical Analyses

Demographics, clinical characteristics, comorbidities, laboratory tests, severity of COVID-19 at admission, treatments, and in-hospital outcomes were compared between COVID-19 patients with and without GI symptoms. Continuous variables were expressed as mean ± standard deviation and median (range), and non-parametric Mann-Whitney *U*-test was used for comparative analyses. Categorical variables were expressed as frequency (percentage), and chi-square and fisher exact tests were used for comparative analyses. Univariate and multivariate logistic regression analyses were performed to evaluate the association of GI symptoms with the composite endpoint and death in COVID-19 patients. Odds ratios (ORs) with 95% confidence intervals (CIs) were calculated. Subgroups analyses were performed in patients with severe/critical COVID-19. A two-tailed *P* < 0.05 was considered statistically significant. All statistical analyses were performed by using IBM SPSS software version 20.0 (IBM Corp, Armonk, NY, USA).

## Results

### Patient Characteristics

After screening, 489 patients were excluded, because 201 patients had hepatobiliary diseases, 136 had a recent history of GI diseases or symptoms before admission, 116 had a history of abdominal surgery, 23 had nephrolithiasis, and 13 were lacking of major clinical data. Finally, 2,552 patients were included.

The characteristics of patients in our study were described in Table 1. The median age was 57.8 years old (range: 11–100) and 50.4% (1,287/2,552) were male. The most common symptoms were fever (72.8%, 1,857/2,552), cough (69.5%, 1,774/2,552), and fatigue and/or myalgia (56.0%, 1,430/2,552). Cardiovascular disease (35.0%, 894/2,552) was the most common comorbidity. At admission, 1.1% (28/2,552), 71.6% (1,827/2,552), 25.8% (659/2,552), and 1.5% (38/2,552) of patients were classified as mild, moderate, severe, and critical COVID-19, respectively.

**Table 1:** Characteristics of COVID-19 patients with and without GI symptoms.

**Variables**	**Overall**		**With GI symptoms**		**Without GI symptoms**		***P*** **-value**
	**No. Pts**	**Median (range) or frequency (percentage);** **Mean ± SD**	**No. Pts**	**Median (range) or frequency (percentage);** **Mean ± SD**	**No. Pts**	**Median (range) or frequency (percentage);** **Mean ± SD**	
Age (>60 years)	2,552	1,227 (48.1%)	537	298 (55.5%)	2,015	929 (46.1%)	***<0.001***
Male (%)	2,552	1,287 (50.4%)	537	243 (45.3%)	2,015	1,044 (51.8%)	***0.007***
**COVID-19 symptoms**							
Fever (%)	2,552	1,857 (72.8%)	537	393 (73.2%)	2,015	1,464 (72.7%)	0.807
Cough (%)	2,552	1,774 (69.5%)	537	365 (68.0%)	2,015	1,409 (69.9%)	0.382
Fatigue and/or myalgia (%)	2,552	1,430 (56.0%)	537	315 (58.7%)	2,015	1,115 (55.3%)	0.168
Dyspnea (%)	2,552	850 (33.3%)	537	190 (35.4%)	2,015	660 (32.8%)	0.251
Chest distress and/or shortness of breath (%)	2,552	583 (22.8%)	537	150 (27.9%)	2,015	433 (21.5%)	***0.002***
Expectoration (%)	2,552	293 (11.5%)	537	68 (12.7%)	2,015	225 (11.2%)	0.334
**Comorbidities**							
Diabetes (%)	2,552	371 (14.5%)	537	85 (15.8%)	2,015	286 (14.2%)	0.339
Cardiovascular disease (%)	2,552	894 (35.0%)	537	205 (38.2%)	2,015	689 (34.2%)	0.086
Cerebrovascular disease (%)	2,552	115 (4.5%)	537	25 (4.5%)	2,015	90 (4.5%)	0.851
Chronic renal disease (%)	2,552	34 (1.3%)	537	11 (2.0%)	2,015	23 (1.1%)	0.103
Chronic respiratory disease (%)	2,552	118 (4.6%)	537	22 (4.1%)	2,015	96 (4.8%)	0.513
Malignant tumor (%)	2,552	56 (2.2%)	537	7 (1.3%)	2,015	49 (2.4%)	0.113
**Laboratory tests**							
Hb (g/L)	2,018	124.45 ± 18.19 124.00 (42.00–318.00)	406	122.05 ± 20.88 123.00 (49.00–318.00)	1,612	125.06 ± 17.41 125.00 (68.00–267.00)	***0.003***
WBC (10^9^/L)	2,020	6.17 ± 2.54 5.70 (1.70–49.30)	406	6.24 ± 2.81 5.75 (2.40–34.10)	1,614	6.15 ± 2.47 5.70 (1.70–49.30)	0.862
PLT (10^9^/L)	2,016	229.84 ± 76.15 220 (6.00–662.00)	406	229.71 ± 70.85 222.00 (48.00–483.00)	1,610	229.88 ± 77.45 219.00 (6.00–622.00)	0.493
TBIL (μmol/L)	1,883	10.83 ± 5.72 9.80 (2.40–112.20)	371	10.93 ± 7.10 9.70 (2.40–112.20)	1,512	10.81 ± 5.33 9.80 (2.40–72.30)	0.906
AST (IU/L)	1,885	23.57 ± 17.73 19.00 (6.10–310.40)	371	25.12 ± 23.69 19.00 (6.40–310.40)	1,514	23.19 ± 15.92 19.05 (6.10–234.50)	0.677
ALT (IU/L)	1,883	29.78 ± 28.76 21.30 (1.70–403.00)	371	28.40 ± 26.35 20.00 (1.70–245.40)	1,512	30.13 ± 29.31 21.40 (4.50–403.00)	0.157
AKP (IU/L)	1,883	74.14 ± 27.29 69.60 (6.00–493.30)	371	73.32 ± 33.74 67.70 (6.00–493.30)	1,512	74.34 ± 25.46 70.35 (16.60–274.60)	0.067
GGT (IU/L)	1,883	40.66 ± 38.02 28.60 (5.40–427.40)	371	38.72 ± 38.35 27.80 (7.90–427.40)	1,512	41.14 ± 37.94 28.80 (5.40–336.60)	0.314
Albumin (g/L)	1,883	38.28 ± 4.10 38.60 (16.40–60.00)	371	37.16 ± 4.48 37.80 (16.40–47.10)	1,512	38.55 ± 3.95 38.80 (22.00–60.00)	*** <0.001***
D-dimer (mg/L)	1,644	0.83 ± 1.88 0.37 (0.01–40.00)	303	0.99 ± 1.70 0.44 (0.01–15.34)	1,341	0.79 ± 1.92 0.36 (0.01–40.00)	***0.015***
PT (seconds)	1,629	12.97 ± 1.24 12.81 (9.20–24.36)	303	12.92 ± 1.28 12.76 (10.20–22.32)	1,326	12.99 ± 1.24 12.81 (9.20–24.36)	0.219
**Severity of COVID-19 at admission**							
Mild type	2,552	28 (1.1%)	537	5 (0.9%)	2,015	23 (1.1%)	0.678
Moderate type	2,552	1,827 (71.6%)	537	352 (65.5%)	2,015	1,475 (73.2%)	*** <0.001***
Severe type	2,552	659 (25.8%)	537	165 (30.7%)	2,015	494 (24.5%)	***0.003***
Critical type	2,552	38 (1.5%)	537	15 (2.8%)	2,015	23 (1.1%)	***0.005***

*COVID-19, coronavirus disease 2019; GI, gastrointestinal; Hb, hemoglobin; WBC, white blood cell; PLT, platelet count; TBIL, total bilirubin; AST, aspartate aminotransferase; ALT, alanine aminotransferase; AKP, alkaline phosphatase; GGT, gamma-glutamyl transpeptidase; PT, prothrombin time*.

*Bold value means there is a statistical difference between the two groups*.

### Prevalence of GI Symptoms in COVID-19 Patients

**Figure 1:**
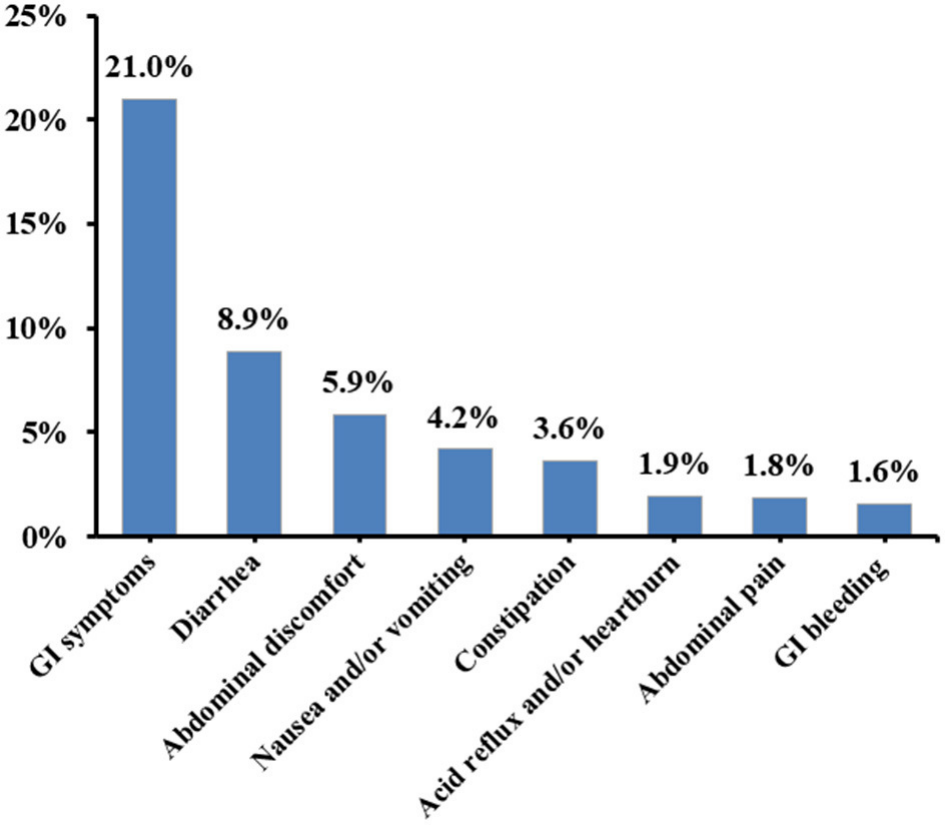
Prevalence of various GI symptoms in COVID-19 patients.

Overall, 21.0% (537/2,552) of COVID-19 patients had at least one type of GI symptom. Diarrhea (8.9%, 226/2,552) was the most common type of GI symptoms, followed by abdominal discomfort (5.9%, 150/2,552), nausea and/or vomiting (4.2%, 106/2,552), constipation (3.6%, 93/2,552), acid reflux and/or heartburn (1.9%, 49/2,552), abdominal pain (1.8%, 47/2,552), and GI bleeding (1.6%, 40/2,552) (Figure 1).

### Difference in Baseline Characteristics Between COVID-19 Patients With and Without GI Symptoms

Patients with GI symptoms had a significantly higher proportion of age > 60 years (55.5 vs. 46.1%; *P* < 0.001) and a lower proportion of male (45.3 vs. 51.8%; *P* = 0.007) than those without (Table 1). Patients with GI symptoms had a significantly higher proportion of chest distress and/or shortness of breath (27.9 vs. 21.5%; *P* = 0.002) than those without. Patients with GI symptoms had a significantly higher d-dimer (0.99 ± 1.70 vs. 0.79 ± 1.92; *P* = 0.015) and lower Hb (122.05 ± 20.88 vs. 125.06 ± 17.41; *P* = 0.003) and albumin (37.16 ± 4.48 vs. 38.55 ± 3.95; *P* < 0.001) than those without. Patients with GI symptoms had a significantly higher proportion of severe/critical COVID-19 at admission (33.5 vs. 25.7%; *P* < 0.001) than those without. The proportion of comorbidity was not significantly different between patients with and without GI symptoms.

### Difference in Treatment Strategy Between COVID-19 Patients With and Without GI Symptoms

Patients with GI symptoms had significantly higher proportions of use of antivirals (57.9 vs. 50.3%; *P* = 0.002), antibiotics (42.3 vs. 30.1%; *P* < 0.001), corticosteroids (21.4 vs. 13.3%; *P* < 0.001), and ECMO/CRRT (1.5 vs. 0.4%; *P* = 0.008) than those without (Table 2).

**Table 2:** Treatment and outcomes of COVID-19 patients with and without GI symptoms.

**Variables**	**Overall**		**With GI symptoms**		**Without GI symptoms**		***P*** **-value**
	**No. Pts**	**Median (range) or frequency (percentage);Mean ± SD**	**No. Pts**	**Median (range) or frequency (percentage);Mean ± SD**	**No. Pts**	**Median (range) or frequency (percentage);Mean ± SD**	
**COVID-19 treatments during hospitalization**							
Antivirals	2,552	1,325 (51.9%)	537	311 (57.9%)	2,015	1,014 (50.3%)	***0.002***
Antibiotics	2,552	834 (32.7%)	537	227 (42.3%)	2,015	607 (30.1%)	***<0.001***
Corticosteroids	2,552	382 (15.0%)	537	115 (21.4%)	2,015	267 (13.3%)	***<0.001***
Traditional Chinese medicines	2,552	2,131 (83.5%)	537	453 (84.4%)	2,015	1,678 (83.3%)	0.548
ECMO/CRRT	2,552	17 (0.7%)	537	8 (1.5%)	2,015	9 (0.4%)	***0.008***
Hospital length of stay	2,252	14.85 ± 8.84	537	18.16 ± 10.62	2,015	13.97 ± 8.07	***<0.001***
(days)		13.00 (0–62)		16.00 (2–61)		12.00 (0–62)	
**Outcomes**							
ICU	2,552	83 (3.3%)	537	32 (6.0)%	2,015	51 (2.5%)	***<0.001***
Mechanical ventilation	2,552	72 (2.8%)	537	26 (4.8%)	2,015	46 (2.3%)	***0.001***
Death	2,552	53 (2.1%)	537	19 (3.5%)	2,015	34 (1.7%)	***0.008***
Composite endpoint	2,552	102 (4.0%)	537	39 (7.3%)	2,015	63 (3.1%)	***<0.001***

*COVID-19, coronavirus disease 2019; ECMO, extracorporeal membrane oxygenation; CRRT, continuous renal replacement therapy; ICU, intensive care unit*.

*Bold value means there is a statistical difference between the two groups*.

### Difference in Outcomes Between COVID-19 Patients With and Without GI Symptoms

**Figure 2:**
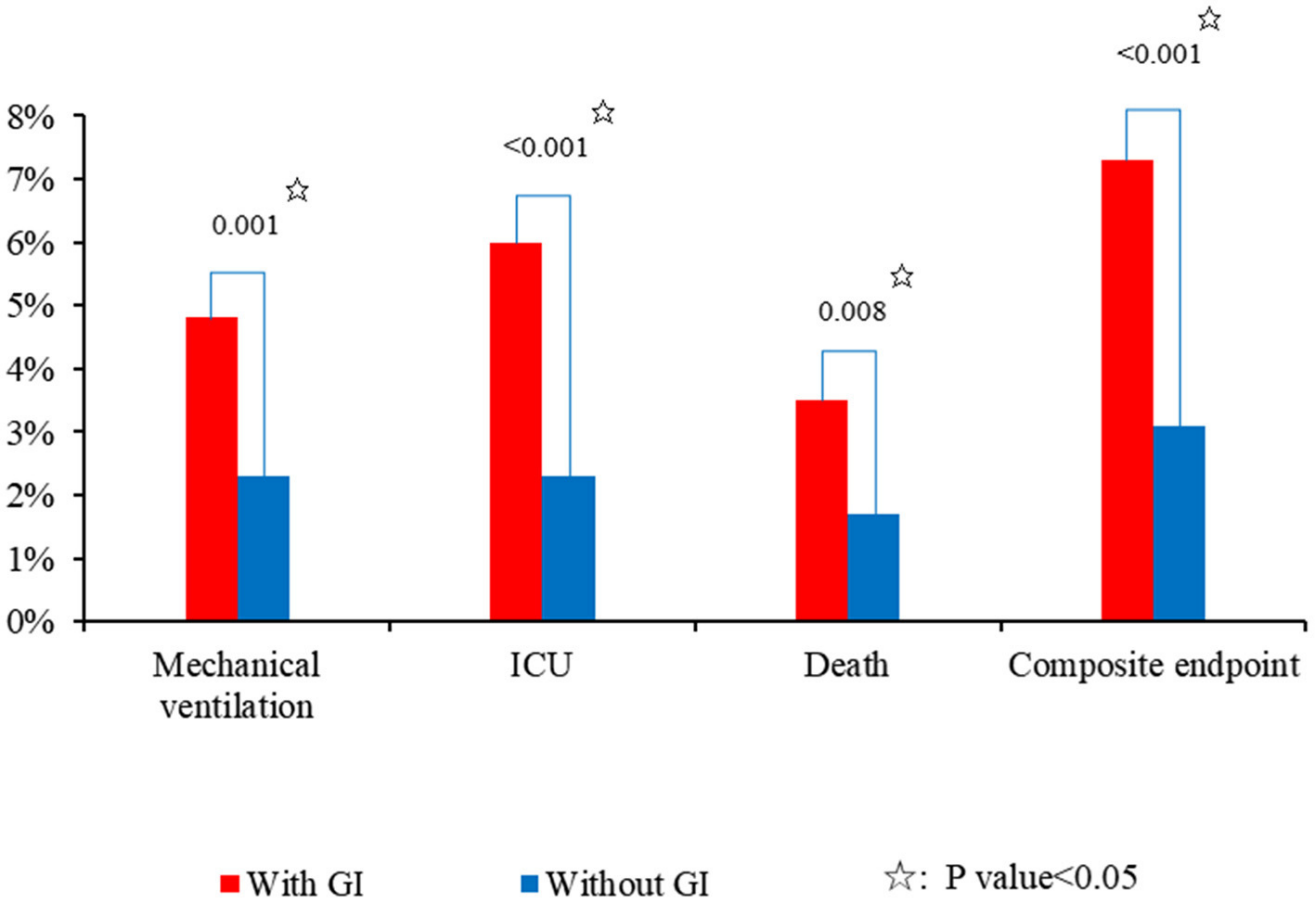
Incidence of adverse outcomes in COVID-19 patients with and without GI symptoms.

Patients with GI symptoms had a significantly longer hospital length of stay (18.16 ± 10.62 vs. 13.97 ± 8.07; *P* < 0.001) than those without (Table 2). Patients with GI symptoms had significantly higher proportions of transferring to ICU (6.0 vs. 2.5%; *P* < 0.001), requiring mechanical ventilation (4.8 vs. 2.3%; *P* = 0.001), death (3.5 vs. 1.7%; *P* = 0.008), and reaching the composite endpoint (7.3 vs. 3.1%; *P* < 0.001) than those without (Figure 2).

### GI Symptoms for Predicting the Composite Endpoint

Univariate logistic regression analyses demonstrated that GI symptoms (OR = 2.426, 95% CI = 1.608–3.661; *P* < 0.001) were significantly associated with the composite endpoint. After adjusting for age, sex, and severe/critical COVID-19, GI symptoms remained an independent predictor of the composite endpoint (OR = 2.029, 95% CI = 1.294–3.182; *P* = 0.002).

In the subgroup analysis of severe/critical COVID-19 patients, univariate logistic regression analyses demonstrated that GI symptoms (OR = 1.941, 95% CI = 1.231–3.058; *P* = 0.004) were significantly associated with the composite endpoint. After adjusting for age and sex, GI symptoms remained an independent predictor of the composite endpoint (OR = 1.950, 95% CI = 1.220–3.115; *P* = 0.005).

According to the type of GI symptoms, univariate logistic regression analyses demonstrated that GI bleeding (OR = 13.108, 95% CI = 6.544–26.255, *P* < 0.001) and abdominal discomfort (OR = 2.010, 95% CI = 1.051–3.844, *P* = 0.035) were significantly associated with the composite endpoint, but not diarrhea, nausea and/or vomiting, constipation, acid reflux and/or heartburn, or abdominal pain. After adjusting for age, sex, and severe/critical COVID-19, only GI bleeding (OR = 8.416, 95% CI = 3.465–20.438, *P* < 0.001), but not abdominal discomfort (OR = 1.262, 95% CI = 0.627–2.542, *P* = 0.514), remained an independent predictor of the composite endpoint.

### GI Symptoms for Predicting Death

Univariate logistic regression analyses demonstrated that GI symptoms (OR = 2.137, 95% CI = 1.209–3.778; *P* = 0.009) were significantly associated with death. After adjusting for age, sex, and severe/critical COVID-19, GI symptoms were not independently associated with death (OR = 1.726, 95% CI = 0.946–3.150; *P* = 0.075).

In the subgroup analysis of severe/critical COVID-19 patients, univariate logistic regression analyses demonstrated that GI symptoms were not significantly associated with death (OR = 1.431, 95% CI = 0.768–2.667; *P* = 0.259).

According to the type of GI symptoms, univariate logistic regression analyses demonstrated that only GI bleeding was significantly associated with death (OR = 13.706, 95% CI = 5.983–31.398, *P* < 0.001), but not diarrhea, abdominal discomfort, nausea and/or vomiting, constipation, acid reflux and/or heartburn, or abdominal pain. After adjusting for age, sex, and severe/critical COVID-19, only GI bleeding (OR = 6.640, 95% CI = 2.567–17.179, *P* < 0.001) remained an independent predictor of death.

## Discussion

The first finding of our study was that 21% of COVID-19 patients had at least one type of GI symptoms during the disease course, which was similar to the data reported by a previous meta-analysis. Notably, the previous meta-analysis also found that the prevalence of GI symptoms was lower in China than in other countries (16 vs. 33%) (12). Indeed, more recent studies from Western countries also found a higher prevalence of GI symptoms (33–61%) (5, 8, 9, 13–15). There are several potential explanations for this phenomenon. First, there are regional and ethnic differences among countries. Second, the definition of GI symptoms is different among studies. Some studies defined anorexia as one type of GI symptoms (5, 13), but others did not (8, 9, 14, 15). From our perspectives, it is more likely that anorexia is a consequence of systemic inflammation rather than a true GI symptom (16). Third, SARS-CoV-2 is being constantly mutated. Different types of mutated virus may lead to a heterogeneity in the prevalence of GI symptoms (17, 18).

There are two major mechanisms regarding the development of GI symptoms in COVID-19 patients. The first one is the SARS CoV-2 S protein-ACE2-TMPRSS2 infection theory. SARS-CoV-2 S protein, which facilitates viral entry into target cells, contains two parts S1 and S2. The function of S1 is to bind the virus to the receptor on the surface of the host cell, and that of S2 is to mediate membrane fusion between the virus and the cell. Human angiotensin-converting enzyme 2 (ACE2), which is the receptor of SARS-CoV-2, binds to the S1 part of SARS-CoV-2 S protein, allowing the virus to attach to the target cell surface. Subsequently, transmembrane serine protease 2 (TMPRSS2), which is mainly located on the surface of the host cell membrane, acts as a primer for the SARS-CoV-2 S protein to activate the S2 part of SARS-CoV-2 S protein, allowing membrane fusion between the virus and the cell. Thus, SARS-CoV-2 enters the host cell (19, 20). In a recent single-cell transcriptomic study, ACE2 and TMPRSS2 were co-expressed in lung, esophagus, ileum, and colon cells, suggesting the possibility of direct viral infection in the digestive system, thereby causing GI symptoms (21). The second one is the lung-gut axis theory. Lung and gut can interact (22). An imbalance of respiratory tract flora can affect the GI tract through the immune system regulation; similarly, a change in the composition and function of GI tract flora can also affect the respiratory tract through the mucosal immune system (23, 24). SARS-CoV-2 may affect the composition of GI tract microbiota through lung infections, thereby causing GI symptoms.

Another major finding of our study was that the presence of GI symptoms significantly increased the risk of the composite endpoint and death. After adjusting for confounding factors, the presence of GI symptoms was still an independent predictor of the composite endpoint, but not death, which indicated that the influence of GI symptoms on the outcomes may not be as strong as the severity of COVID-19. Two previous meta-analyses found that GI symptoms were not associated with death, but they did not include GI bleeding as one type of GI symptoms (25, 26). Our study further evaluated whether various types of GI symptoms were associated with the composite endpoint and death, and found that GI bleeding was an independent predictor of the composite endpoint and death. The association of GI symptoms with worse outcomes can be explained by the following considerations. First, the contribution of GI symptoms on the deterioration of outcomes in COVID-19 patients should be primarily attributed to the development of GI bleeding, which is far more lethal than other GI symptoms. GI bleeding is commonly associated with critical illness (27). Acute massive GI bleeding can cause unstable hemodynamics, leading to shock and even death (28). Chronic occult GI bleeding may cause anemia (29). When there is a significantly decreased concentration of hemoglobin, a carrier of oxygen, the transportation of oxygen to various organs will be interrupted, causing organic hypoxia and then multiple organ dysfunction (30). Second, GI symptoms are more prone to develop electrolyte disturbances (4). Third, patients with GI involvement have a higher viral load and/or more prolonged viral shedding (31). Fourth, small intestine is the human body's largest immune organ. SARS-CoV-2 can directly infect small intestine and cause its immune dysfunction, which may enhance or even drive systemic inflammatory response (32). Fifth, some drugs that are commonly used to treat GI symptoms, such as proton pump inhibitors, may increase the severity of COVID-19 and the risk of worse outcomes (33). Sixth, GI symptoms as the first clinical manifestation in some COVID-19 patients may delay the diagnosis and treatment until it has progressed to more advanced stage (34). In our study, patients with GI symptoms had a longer hospital length of stay and received antivirals, antibiotics, and corticosteroids more frequently during their hospitalizations than those without, which suggests that patients with GI symptoms had more severe COVID-19.

Our study has several major features. First, we had a large number of COVID-19 patients consecutively hospitalized at the Wuhan Huoshenshan hospital during the same period. Second, we excluded the conditions that may cause GI symptoms before SARS-CoV-2 infection, such as hepatobiliary diseases, nephrolithiasis, and history of GI diseases or symptoms and abdominal surgery. Third, the GI symptoms mainly evaluated in previous studies were abdominal pain, diarrhea, nausea, and vomiting. By comparison, we further included abdominal discomfort, constipation, acid reflux and/or heartburn, and GI bleeding. Fourth, we performed multivariate analyses to explore the association of GI symptoms with the composite endpoint and death by adjusting for age, sex, and severe/critical COVID-19.

Our study also has several limitations. First, this was a retrospective study where not all GI symptoms had been sufficiently recorded, probably underestimating the prevalence of GI symptoms. Second, our study could not evaluate the severity and duration of GI symptoms. Third, some GI symptoms might not be caused by SARS-CoV-2 infection. It was difficult to judge the nature of GI symptoms. Fourth, our current data reflected the disease condition and its effects at the beginning of the SARS-CoV-2 outbreak in China, but not the outcomes caused by more recently mutated SARS-CoV-2. Fifth, because the Wuhan Huoshenshan hospital had been closed since the epidemic was effectively controlled in April 2020, we cannot continue to collect more new data on patients with COVID-19 from this hospital.

In conclusion, GI symptoms are common in COVID-19 patients and may be associated with worse outcomes. Notably, the impact of GI symptoms on the outcomes should be due to GI bleeding, but not other GI symptoms. When COVID-19 patients have or develop GI bleeding, clinicians should be alert to a higher risk of disease progression and death. In future studies, it is necessary to prospectively and systematically collect the GI symptoms in COVID-19 patients at admission and during hospitalization, and to further explore the association of GI symptoms with prognosis.

## Data Availability Statement

The original contributions presented in the study are included in the article/supplementary material, further inquiries can be directed to the corresponding authors.

## Ethics Statement

The studies involving human participants were reviewed and approved by the Medical Ethical Committee of the General Hospital of Northern Theater Command. Written informed consent from the participants' legal guardian/next of kin was not required to participate in this study in accordance with the national legislation and the institutional requirements.

## Author Contributions

XQ: conceptualization. HC, LL, and XQ: formal analysis. HC, ZT, ZM, LL, YTa, YTe, HY, HM, CP, QZ, TZ, GC, HLi, HLu, and XQ: data curation. HC and XQ: writing—original draft. HC, ZT, ZM, YTa, YTe, HY, HM, CP, QZ, TZ, HZ, GC, HLi, HLu, and XQ: writing—review and editing. HLi, HLu, and XQ: supervision. All authors have made an intellectual contribution to the manuscript and approved the submission.

## Conflict of Interest

The authors declare that the research was conducted in the absence of any commercial or financial relationships that could be construed as a potential conflict of interest.

## Publisher's Note

All claims expressed in this article are solely those of the authors and do not necessarily represent those of their affiliated organizations, or those of the publisher, the editors and the reviewers. Any product that may be evaluated in this article, or claim that may be made by its manufacturer, is not guaranteed or endorsed by the publisher.

## Footnotes

^1^ https://www.who.int/emergencies/diseases/novel-coronavirus-2019

## Abbreviations

SARS-CoV-2: severe acute respiratory syndrome coronavirus 2
COVID-19: cause coronavirus disease 2019
GI: gastrointestinal
Hb: hemoglobin
WBC: white blood cells
PLT: platelet
TBTL: total bilirubin
AST: aspartate aminotransferase
ALT: alanine aminotransferase
AKP: alkaline phosphatase
GGT: gamma glutamyl transpeptidase
PT: prothrombin time
ECMO: extracorporeal membrane oxygenation
CRRT: continuous renal replacement therapy
ICU: intensive care unit
SpO_2_: oxygen saturation
PaO_2_: arterial partial pressure of oxygen
FiO_2_: fraction of inspiration oxygen
ORs: odds ratios
CIs: confidence intervals
ACE2: angiotensin-converting enzyme 2
TMPRSS2: transmembrane serine protease 2.
